# Effectiveness of the NOLA Dry Field device as an isolation system for bonding lower lingual retainers

**DOI:** 10.4317/jced.63055

**Published:** 2025-09-01

**Authors:** Sara Antelo-Ríos, Henar Sastre-Álvaro, Iván Nieto-Sánchez, Laura del Campo-Albendea

**Affiliations:** 1 Orthodontic Graduate student. Centro Odontológico Hospital San Rafael. Francisco de Vitoria University. Madrid; 2 Orthodontic Graduate Lecturer. Centro Odontológico Hospital San Rafael. University Francisco de Vitoria; 3 Biostatiscian. Ramón y Cajal University Hospital – Francisco de Vitoria University

## Abstract

**Background:**

The retention stage has been the most controversial phase of orthodontic treatment, as there are no specific guidelines regarding the type, mode and duration of retention. The detachment of a retainer creates urgency, necessitates additional consultation time and increases the risk of tooth movement. Therefore, ensuring the stability of a retainer and preventing detachment is crucial, particularly as saliva moisture poses a risk during bonding.

**Material and Methods:**

This pilot study employs an epidemiological, observational, descriptive and longitudinal design to compare the failure rates of lower lingual retainers cemented with the NOLA retractor against those cemented with the Spandex retractor. The sample comprised 32 participants, with 16 each in the NOLA group and the relative isolation group. The NOLA group included 10 men (62.5%), while the isolation group had only 3 men (18.7%). The normality of the data was assessed using the Shapiro–Wilk test, and the Student’s t-test was applied to compare changes between the NOLA and relative isolation groups.

**Results:**

The failure rate was higher in the relative isolation group (37.5%) compared with the NOLA group (25.0%), although this difference was not statistically significant (*p* = 0.446). A progressive and significant increase in the Little’s Irregularity Index values was observed in both groups over time. While there was a greater increase in values in the NOLA group, the difference did not reach statistical significance (*p* = 0.108 at 3 months and *p* = 0.284 at 6 months).

**Conclusions:**

Both isolation methods demonstrated similar success rates in retaining fixed retainers over the analysed time period.

** Key words:**Orthodontic Retainers, Fixed Orthodontic Appliance, Removable Orthodontic Appliances, Periodontal Index.

## Introduction

In 1934, Oppenheim stated that ‘retention is one of the most difficult problems in orthodontics; in fact, it is the problem’ [1-3]. To mitigate relapse, various orthodontic retainers are employed to keep teeth in their correct positions following orthodontic treatment [4].

For a long time, the retention phase has been generating considerable debate due to the absence of specific or widely accepted standards regarding the type, mode and duration of retention [5]. The detachment of a retainer creates urgency, necessitates additional consultation time and increases the risk of tooth movement.

Retention is essential for several reasons, the most significant of which are as follows:

1. The periodontal tissues surrounding the teeth undergo alterations following orthodontic movement and require time to reorganise [1,6].

2. The soft tissue pressures within the oral cavity can exert constant forces, leading to a tendency for relapse if teeth are left in an unsTable position [1,6].

3. Growth may result in changes that could affect the quality and outcome of orthodontic treatment [1,6].

Adhesion of a fixed retainer to the six anterior inferior teeth provides greater stability, which is the focus of our study.

Fixed retainers offer advantages such as improved aesthetics, durability and reduced reliance on patient cooperation; however, they tend to have a higher failure rate than removable retainers [7-9].

Adhesion of a fixed retainer

A fixed retainer can be bonded using two different isolation systems: Spandex and NOLA.

**Figure 1:**
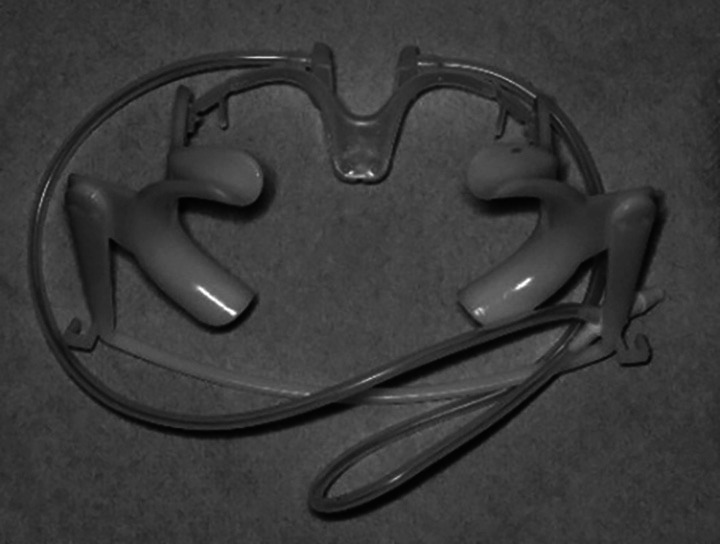
NOLA isolation system.

NOLA isolation system: This system includes an adjusTable cheek separator, a saliva ejector, a tongue guard and a low-volume suction connection (Fig. 1).

**Figure 2:**
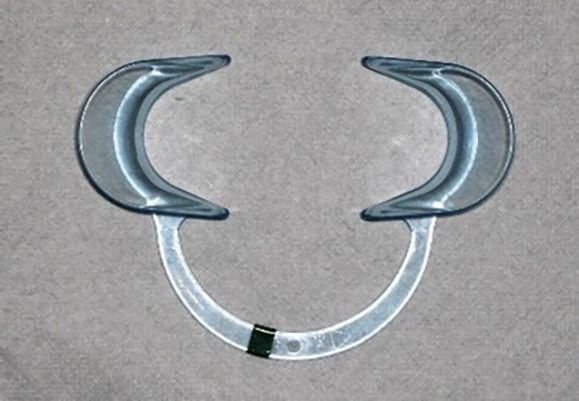
Spandex isolation system.

Spandex isolation system: This system is made of anti-reflective plastic and, unlike the NOLA isolation system, lacks a saliva ejector, tongue guard and low-volume suction connection (Fig. 2).

## Material and Methods

- Study design

**Figure 3:**
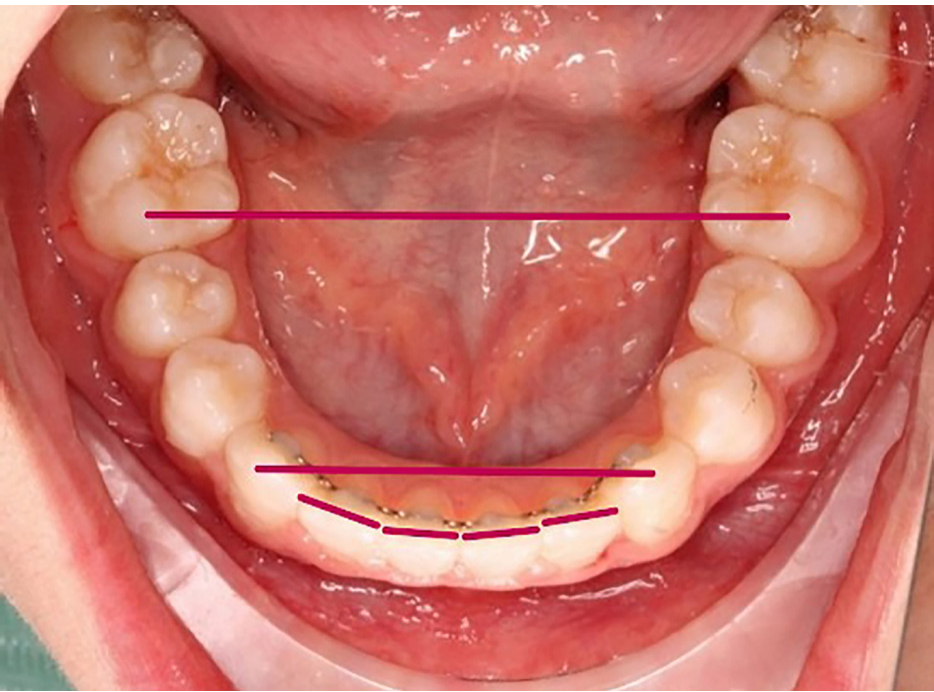
Parameters measured in our study: intermolar width, intercanine width and Little’s Irregularity Index.

The present study is a pilot, epidemiological, observational, descriptive and longitudinal investigation comparing lower lingual retainers cemented with the NOLA retractor against those cemented with the Spandex retractor (relative isolation). The degree of relapse following orthodontic treatment was measured using intercanine width, intermolar width and the Little’s Irregularity Index. Intercanine width is defined as the distance between the cusp tips of the right and left canines, while intermolar width is the distance between the tips of the mesiobuccal cusps of the left and right lower first molars [10]. The Little’s Irregularity Index measures the linear displacement of the anatomical contact points of the lower incisors. Measurements were performed between the lower canines, and the displacement values were summed up to obtain the Irregularity Index [11], (Fig. 3).

The target population comprised patients of both sexes, aged between 14 and 50 years, treated in the Odontostomatology Department of the Dental Center of Hospital San Rafael from November 2023 to December 2024.

Initially, patients (or parents/guardians in case of minors) were informed and provided with a study information sheet. Subsequently, they signed the informed consent form. The study adhered to the Helsinki criteria and all current data protection regulations and received approval from the San Juan de Dios Research Commission (protocol number: No. P_TFM_O_2024_003).

Data were collected and pseudonymized from subjects who had fixed lower retainer bonded, following their voluntary agreement to participate and the signing of the consent form.

Inclusion criteria encompassed children and adults undergoing orthodontic treatment with clear aligners, conventional brackets or aesthetic brackets, completed at the Dental Center of Hospital San Rafael.

Exclusion criteria comprised patients who did not complete the informed consent form, were syndromic, had bone pathology or were on medication that could influence dental movement (e.g. bisphosphonates), and had xerostomia.

Data were collected from a total of 32 patients, divided into two groups: 16 patients who had retainers placed with the NOLA retractor and 16 patients who had retainers placed with the Spandex retractor. This division aimed to analyse the occlusal relapse rate and failure rate between both groups concerning the placement of lower lingual retainers.

The primary variable was the recurrence rate of lower incisor and canine alignment in both groups.

Other variables studied included the duration of the cementing appointment, number of emergency appointments between scheduled visits and the frequency of retainer breakage or detachment. Sociodemographic variables such as sex, age and years of clinical practice of the operators were also recorded.

Patients were randomly assigned (https://www.randomizer.org/) into the two groups: group 1 comprised patients who had the retainer fitted with the NOLA retractor (N = 16), while group 2 included patients fitted with the lingual lower fixed retainer using the Spandex retractor (N = 16). An intraoral scan was performed on the day of orthodontic appliance removal for both groups.

Patients were reviewed at three and six months. At these appointments, a scan of both arches was performed, and data on the number of emergencies between appointments, points of retainer detachment or instances of retainer breakage were collected.

After an exhaustive literature review, the following bonding protocol was determined for fixed retainers [14]:

1) After debonding the orthodontic appliances, the lingual surfaces of the six anterior inferior teeth were cleaned and isolated.

2) Each tooth was treated according to the manufacturer’s instructions, including etching with 37% phosphoric acid gel for 30 seconds, followed by rinsing, drying of the tooth surface, and bonding with a light-curing adhesive using a Woodpecker wireless light-curing light with a power of 1000 mW/cm² for 20 seconds.

3) Subsequently, strings of dental floss were placed in each interproximal space from canine to canine.

4) The wire was then positioned over the teeth and secured with the previously placed dental floss.

5) The wire was subsequently attached to the lingual surfaces of the teeth and bonded using the specified composite for 40 seconds.

6) Finally, the surface was checked for smoothness, and any remaining premature contacts were removed.

- Statistical analysis

A descriptive analysis of the data was conducted, presenting quantitative variables as means, standard deviations (SDs), medians and quartiles (Q1; Q3), as well as minimum and maximum values. Qualitative variables were expressed as absolute frequencies and percentages (%).

The normality of the data for intermolar distance (IMD), intercanine distance (ICD) and Little’s Irregularity Index (LII) was evaluated using the Shapiro–Wilk test, which enabled the selection of the most appropriate statistical tests for comparison between the groups. To assess the efficacy of each treatment, the change from baseline was calculated within each group. Subsequently, the Student’s t-test was applied to compare the changes between the NOLA and relative isolation groups. Additionally, as a secondary analysis, we evaluated whether significant differences existed between baseline values and the values obtained at 3 and 6 months within each group for each of the parameters analysed.

The chi-square test was employed to analyse the possible association of the failure results and the NOLA . All statistical analyses were performed with a significance level of *p* < 0.05. Analyses were conducted using R 4.3.1. [12].

## Results

Description of the sample

The analysed sample consisted of 32 participants, with 16 in the NOLA group (50.0%) and 16 (50.0%) in the relative isolation group (Spandex). The NOLA group included 10 male (62.5%) and 6 female (37,5%) , while the isolation group had only 3 men (18.7%) and 13 female (81,3%) .

Description of measured parameters and comparison between groups

 Table 1 presents the values of the three parameters evaluated: intermolar distance (IMD), intercanine distance (ICD) and Little’s Irregularity Index (LII) at various follow-up times (on the day of orthodontic appliance removal, at 3 months and at 6 months) for both groups.

• IMD (intermolar distance): The mean values in the NOLA group were slightly higher than those in the relative isolation group at all time points, but without significant changes in the mean at follow-up.

• ICD (intercanine distance): Values remained sTable in both groups, with minimal differences between measurements.

• LII (Little’s Irregularity Index): A progressive increase was observed in both groups over time, suggesting an increase in dental irregularity, although the change was greater in the NOLA group.

The dispersion values (SD and interquartile ranges) reflected similar variability between the groups.

 Table 2 presents the differences in the parameters measured at various follow-up times and the *p-value*s for comparisons between the groups.

• IMD (intermolar distance): No statistically significant differences were found between the NOLA and relative isolation groups at any measurement point (*p* > 0.05 in all comparisons). Furthermore, the variation in the mean from the baseline measurement was minimal.

• ICD (intercanine distance): Changes in the mean were almost non-existent in both groups throughout follow-up, and the *p-value*s confirm the absence of significant differences between the groups (*p* > 0.05).

• LII (Little’s Irregularity Index): A progressive and significant increase in LII values was observed in both groups over time. While this increase was greater in the NOLA group, the differences between the groups did not achieve statistical significance (*p* = 0.108 at 3 months and *p* = 0.284 at 6 months).

Overall, the analysis indicated no significant changes in the parameters analysed between the groups or within each group over time.

- Failure rate according to the isolation system

 Table 3 compares the failure rates between fixed retainers placed with NOLA and those placed with relative isolation.

The failure rate was higher in the relative isolation group (37.5%) compared with the NOLA group (25.0%), although this difference was not statistically significant (*p* = 0.446).

Disaggregation of the data were by sex revealed the following:

• Among males, the failure rates were similar in both groups (30.0% in NOLA and 33.3% in relative isolation, *p* = 0.913).

• Among females, the failure rate was higher in the relative isolation group (38.5%) compared with the NOLA group (16.7%), although this difference was not statistically significant (*p* = 0.342).

These results suggest that, while the failure rate appears slightly higher with relative isolation, the difference is not statistically significant in this sample.

## Discussion

As previously noted, the retention phase is critical following orthodontic treatment, representing the final stage of the process. Our analysis revealed that failures with the retainer predominantly occurred within the initial months. This finding aligns with the study by Aye [13] in 2023, which indicated that half of the failures occurred within the first year, with the majority reported within the first six months. This observation was also corroborated by the study conducted by Lyros [14], which found that nearly all patients wearing a fixed lingual retainer exhibited changes in tooth position in the comparison of the initial 3D models with those taken six months after cementation [14].

Regarding intermolar distance (IMD), intercanine distance (ICD) and Little’s Irregularity Index (LII), no significant differences in change were noted in our study. Although the mean values for intermolar spacing were slightly higher with the NOLA isolation system, these differences were not statistically significant. Similarly, there were no significant differences in intercanine width, remaining sTable across different measurements. Finally, regarding the Little’s Irregularity Index, a progressive and significant increase was observed in both groups over time. While the increase in values was greater in the NOLA group, the differences between the groups did not reach statistical significance (*p* = 0.108 at 3 months and *p* = 0.284 at 6 months).

The achievements of this pilot study include its status as the first to compare these two different types of isolation, while its limitations include its investigation in a single centre.

## Conclusions

No significant differences were identified between the groups in terms of intermolar distance, intercanine distance and Little’s Irregularity Index measurements over time. The Little’s Irregularity Index (LII) exhibited an increasing trend over time in both groups. The failure rate was higher in the relative isolation group (37.5%) compared with the NOLA group (25.0%), although this difference was not statistically significant. No differences in failure rates were observed between sexes. These findings suggest that both isolation methods examined have comparable success rates in retaining fixed retainers over the analysed time period.

## Figures and Tables

**Table 1:** Description of the parameters measured at different follow-up times (N = 32).

	Beginning		3 months		6 months	
Group	NOLA Dry Field Device N = 16	Isolation N = 16	NOLA Dry Field Device N = 16	Isolation N = 16	NOLA Dry Field device N = 16	Isolation N = 16
IMD						
Mean (SD)	33.7 (2.4)	32.7 (2.1)	33.6 (2.3)	32.6 (2.1)	33.6 (2.3)	32.5 (2.1)
Median [Q1; Q3]	33.7 [32.2; 35.1]	33.2 [31.1; 34.0]	33.5 [32.0; 35.0]	33.2 [31.0; 33.9]	33.4 [32.1; 35.1]	33.1 [30.9; 33.8]
Min. - Max.	28.9, 38.6	29, 36.5	29.1, 38.6	28.9, 36.4	29, 38.8	28.9, 36.4
ICD						
Mean (SD)	26.9 (2.3)	26.3 (1.1)	26.9 (2.3)	26.3 (1.3)	26.9 (2.5)	26.2 (1.3)
Median [Q1; Q3]	26.8 [25.3; 28.9]	26.2 [25.7; 27.1]	26.7 [25.4; 29.2]	26.1 [25.6; 27.1]	26.7 [25.4; 29.0]	26.0 [25.5; 27.0]
Min. - Max.	22.4, 30.4	24, 28.7	22.4, 29.9	23.7, 29.2	22.3, 30.3	23.5, 29.0
LII						
Mean (SD)	2.4 (0.9)	2.5 (0.8)	2.8 (0.8)	2.7 (0.7)	2.9 (0.8)	2.9 (0.7)
Median [Q1; Q3]	2.1 [1.8; 2.8]	2.2 [2.0; 3.0]	2.5 [2.2; 3.2]	2.4 [2.2; 3.2]	2.9 [2.3; 3.4]	2.6 [2.5; 3.4]
Min. - Max.	1.4, 4.1	1.4, 4.4	1.8, 4.9	1.6, 4.1	1.8, 4.3	1.8, 4.0

**Table 2:** Differences in the parameters measured at different follow-up times (N = 32) between groups and versus their initial time.

	Beginning		3 months			6 months		
Group	NOLA Dry Field device N = 16	Isolation N = 16	NOLA Dry Field device N = 16	Isolation N = 16	p-value	NOLA Dry Field device N = 16	Isolation N = 16	p-value
IMD								
Mean (SD)	33.7 (2.4)	32.7 (2.1)	33.6 (2.3)	32.6 (2.1)	0.505	33.6 (2.3)	32.5 (2.1)	0.924
Change in average	-	-	0.1 [-1.54; 1.83]	0.1 [-1.4; 1.6]		0.2 [-1.5; 1.9]	0.2 [-1.3; 1.7]	
p - significant value with respect to baseline measurement			No sig.	No sig.		No sig.	No sig.	
ICD								
Mean (SD)	26.9 (2.3)	26.3 (1.1)	26.9 (2.3)	26.3 (1.3)	0.623	26.9 (2.5)	26.2 (1.3)	0.531
Change in average	-	-	-0.0 [-0.2; 0.2]	0.1 [-0.1; 0.2]		0.04 [-0.3; 0.4]	0.2 [-0.03; 0.3]	
p - significant value with respect to baseline measurement			No sig.	No sig.		No sig.	No sig.	
LII								
Mean (SD)	2.4 (0.9)	2.5 (0.8)	2.8 (0.8)	2.7 (0.7)	0.108	2.9 (0.8)	2.9 (0.7)	0.284
Change in average	-	-	0.3 [0.1; 0.6]	0.2 [0.1; 0.3]		0.5 [0.2; 0.8]	0.3 [0.2; 0.5]	
p - significant value with respect to baseline measurement			Sig.	Sig.		Sig.	Sig.	

**Table 3:** Comparison of the failure rate between treatment with NOLA versus relative isolation (n = 32).

	NOLA Dry Field device	Isolation	p-value
Total, n	16	16	
Success	12 (75.0%)	10 (62.5%)	0.446
Failure	4 (25.0%)	6 (37.5%)	
Male, n	10	3	
Success	7 (70.0%)	2 (66.7%)	0.913
Failure	3 (30.0%)	1 (33.3%)	
Female, n	6	13	
Success	5 (83.3%)	8 (61.5%)	0.342
Failure	1 (16.7%)	5 (38.5%)	

## Data Availability

The datasets used and/or analyzed during the current study are available from the corresponding author.

## References

[1] Littlewood SJ, Kandasamy S, Huang G (2017). Retention and relapse in clinical practice. Aust Dent J.

[2] Bjering R, Birkeland K, Vandevska-Radunovic V (2015). Anterior tooth alignment: A comparison of orthodontic retention regimens 5 years posttreatment. Angle Orthod.

[3] Gelin E, Seidel L, Bruwier A, Albert A, Charavet C (2020). Innovative customized CAD/CAM nickel-titanium lingual retainer versus standard stainless-steel lingual retainer: A randomized controlled trial. Korean J Orthod.

[4] Andriekute A, Vasiliauskas A, Sidlauskas A (2017). A survey of protocols and trends in orthodontic retention. Prog Orthod.

[5] Popovid Z, Trinajstid Zrinski M, Špalj S (2020). Orthodontist clinical experience and clinical situation significantly influence the retention protocol - A survey from Croatia. Acta Clin Croat.

[6] Littlewood SJ (2017). Responsibilities and retention. APOS Trends Orthod.

[7] Labuneț A, Objelean A, Almășan O, Kui A, Buduru S, Sava S (2022). Bruxism’s implications on fixed orthodontic retainer adhesion. Dent J (Basel).

[8] García-Espona Meléndez ME, García Espona I (2020). Nuevos desarrollos CAD-CAM de retención fija en ortodoncia. Ortod Esp.

[9] Güneş RO, Sayar G, Toygar H (2023). Clinical comparisons of different fixed orthodontic retainers. Dental Press J Orthod.

[10] Garg H, Khatria H, Kaldhari K, Singh K, Purwar P, Rukshana R (2021). Anchura intermolar e intercanina cambios entre las maloclusiones de clase I y clase II después del tratamiento ortodóncico. Int J Clin Pediatr Dent.

[11] Arango C, Lopez MI, Ramirez C, Jimenez V ID (1992). Presion labial en individuos con diferentes grados de irregularidad dental. Revista CES Odontología.

[12] (2016). R: A language and environment for statistical computing. R Core Team.

[13] Aye ST, Liu S, Byrne E, El-Angbawi A (2023). The prevalence of the failure of fixed orthodontic bonded retainers: A systematic review and meta-analysis. Eur J Orthod.

[14] Lyros I, Tsolakis IA, Maroulakos MP, Fora E, Lykogeorgos T, Dalampira M (2023). Orthodontic retainers-A critical review. Children.

